# Comparative Evaluation of Artifacts Originated by Four Different Post Materials Using Different CBCT Settings

**DOI:** 10.3390/tomography8060245

**Published:** 2022-12-13

**Authors:** Dilek Helvacioglu-Yigit, Umut Seki, Sebnem Kursun-Cakmak, Husniye Demirturk Kocasarac, Maharaj Singh

**Affiliations:** 1 College of Dental Medicine, QU Health, Qatar University, Doha 2713, Qatar; 2 Department of Dentomaxillofacial Radiology, Faculty of Dentistry, Kocaeli University, Kocaeli 41190, Turkey; 3 Department of Dentomaxillofacial Radiology, Faculty of Dentistry, Ankara Yıldırım Beyazıt University, Ankara 1487, Turkey; 4 BeamReaders Inc., Kennewick, WA 99336, USA; 5 Department of Dentomaxillofacial Radiology, Kocaeli Health and Technology University, Kocaeli 41000, Turkey; 6 College of Nursing, Marquette University, Milwaukee, WI 53233, USA

**Keywords:** artifacts, beam hardening, cone-beam computed tomography, dental post

## Abstract

The aim of this study was to evaluate whether cone beam computed tomography (CBCT) images in the presence of four different post materials, obtained from different kVps with varying resolutions and varying metal artifact reduction (MAR) algorithms, differed in artifact estimation, and to compare tooth regions in terms of artifact value. Materials and Methods: Forty premolar teeth were used in this study. Root canals were treated, and teeth were randomly distributed into four subgroups (*n* = 10) for the preparation of post materials: titanium, gold (Nordin), quartz fiber (Bisco DT Light), and glass fiber (Rely X). The CBCT images were taken with two different kVps, three different metal artifact reduction (MAR) algorithm options, and two different resolutions. For each protocol, the effective dose was calculated according to the dose area production (DAP) value. The standard analysis of variance technique and the Tukey multiple comparison adjustment method were used to assess interactions among material types, kVp, MAR, and voxel settings. Results: More artifacts were found in the middle third than in the cervical third (*p* < 0.05). The mean value of artifacts was highest for gold (Nordin), 90 kVp, no MAR, and 100 voxel size. Glass or quartz fiber posts at low resolution, with high MAR and 96 kVp, originated fewer artifacts. Moreover, the use of 90 and 96 kVp with 200 voxel size and high MAR provided the least amount of radiation. Conclusion: The best setting for radiographic follow-up of post materials on the Planmeca ProMax is 96 kVp with low resolution and high MAR; this setting produced one of the lowest effective doses. Clinical Significance: This study estimated the best scanning protocol by lowering the effective dose to a minimum level according to the “as low as reasonably achievable” principle, as well as assessing the tooth region and the post material generating the fewest artifacts, in order to prevent image interpretation challenges such as false-positive and false-negative results stemming from the deterioration of the visibility of the root canal due to perforation, fractures, and voids in the root canal region.

## 1. Introduction

Artifacts are caused by inconsistency in the reconstructed data and the existing content of the material due to its properties [1]. The gray-level nonuniformities in CBCT (cone-beam computed tomography) promote image degradation and may cause inaccurate or false diagnoses by obscuring or simulating the pathology [2,3].

CBCT is a proposed technique in endodontic evaluations due to its delicate and precise diagnostic characteristics. Despite a growing trend of CBCT in dentistry, it has some disadvantages such as artifacts. In cases with high-density post materials, various artifacts such as noise, scatter, motion missing values, and rings, as well as beam hardening and streaking, may occur, leading to image interpretation challenges [4].

Beam hardening is one of the most encountered artifacts, which results in dark streaks in CBCT images. Beam hardening is more noticeable on CBCT images due to the heterochromatic nature and lower kVp energy of CBCT X-ray beams compared with medical CT [3].

Recent studies have indicated that CBCT image quality is changed by factors such as kilovoltage peak (kVp), milliamperage (mA), voxel, and field of view (FOV) sizes [4,5,6,7].

The usage of a higher kVp and mAs improves image homogeneity and uniformity compared to the lower kVp and mAs [8]. Voxel size is directly related to the spatial resolution of an image; as voxel size decreases, resolution and detail increase. Images with reduced FOV size exhibit less scatter and fewer artifacts, thus resulting in higher contrast and images with less noise, as well as qualitative improvements in image quality for specific diagnostic tasks [9].

It has been stated that CBCT devices should be optimized by enabling exposure factors that are appropriate for artifact reduction and other image quality parameters, finding a balance with the radiation dose [10,11].

A dental post is required to improve the retention of restoration of endodontically treated teeth with insufficient dental tissue [12]. Dental posts have a variety of types made of different materials. Stainless-steel, titanium, carbon, and cast posts have been used for many years. Recently, fiber posts have gained popularity because of their mechanical properties, easy application, and aesthetic benefits. The chemical composition of dental posts also affects their radiopacity, which is an essential property to visually distinguish them from the hard tissues and to evaluate their adaptation to the canal space [13]. However, this property may limit the diagnostic quality of CBCT imaging during radiologic evaluation [14]. Chemical compositions with high-atomic-number alloys generate more image artifacts, which could hinder and/or mimic the voids, perforations, and fractures in the root canal [15,16].

Eventually, regardless of the image quality with various combinations of these parameters, high-density objects are still degraded in the image quality because of their higher atomic number and higher capacity to absorb X-rays [17].

The goal of this study was to evaluate whether CBCT images, in the presence of four different post materials, obtained from different kVps with varying resolutions and varying metal artifact reduction (MAR) algorithms, differ in artifact estimation, and to compare tooth regions in terms of artifact value.

## 2. Materials and Methods

### 2.1. Sample Selection and Preparation

Approval of the use of extracted human teeth was obtained by the Medical Ethics Committee of Kocaeli University, Kocaeli, Turkey (Review No. KÜ GOKAEK 2017/165). Forty non-carious single-rooted extracted mandibular premolar teeth were selected for this study, and teeth with previous endodontic treatment, open apex, root resorption, fractures, any anomaly, or structural defects were excluded.

Teeth were all decoronated with diamond burs using a high-speed handpiece under water cooling, and the length of the roots was standardized at 12 mm. The root canals were prepared using ProTaper Next X5 (Dentsply/Maillefer, Ballaigues, Switzerland). The root canals were obturated with a lateral compaction of gutta-percha and silver-free AH26 sealer (Dentsply Sirona, Bensheim, Germany). Then, the roots were stored in 100% humidity for 1 week.

Teeth were randomly distributed into four subgroups according to the type of post:

Group 1 (*n* = 10): Titanium alloy post 0.8 mm in diameter (Unimetric Dentsply, Maillefer Ins Holding Ballaigues, Switzerland (Lot: 1356737);

Group 2 (*n* = 10): Prefabricated screw-type gold-plated post medium #1, conical cross (Nordin, Chailly, Switzerland) (Lot: 14995/263);

Group 3 (*n* = 10): Quartz fiber Bisco DT Light Illusion X-Ro Post #1 (Bisco Inc, Schaumburg, IL, USA) (Lot: 1400005973);

Group 4 (*n* = 10): Rely-X fiberglass post #2 (3M ESPE, Neuss, Germany) (Lot: 379631802).

The post drills of each system were used to prepare 8 mm of post space, leaving 3–4 mm of gutta-percha at the apical portion of each root. After gutta-percha removal and post space preparation, prior to post cementation, posts with stoppers were placed into the root canal, and the post space length was confirmed by measuring the post length within the root canal. Posts were than cemented using self-adhesive resin cement (RelyX U200, 3M ESPE, Neuss, Germany).

### 2.2. CBCT Imaging

**Figure 1:**
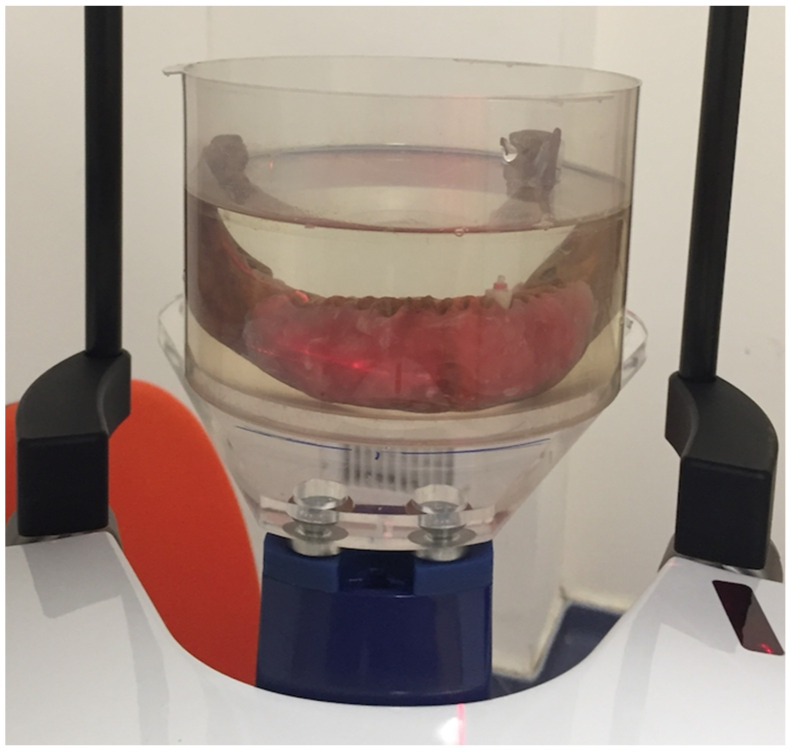
Mandible placed in a round plastic box with wax covering the alveolar crest.

The root sample was placed in an empty anterior socket of a dried mandible taken from the Department of Anatomy, Faculty of Dentistry, Kocaeli University. In order to best simulate alveolar soft tissue during exposure, the mandible was covered with a 4 mm thick pink wax layer, and a round plastic box was filled with water to cover the area of interest. Red markers were used to help replace the mandible in the same position after changing the root (Figure 1).

The CBCT images were taken using a Planmeca CBCT machine (Planmeca ProMax 3D Max) with the following parameters: two different kVps (90 kVp and 96 kVp), three different metal artifact reduction (MAR) algorithms (no MAR, medium MAR, and high MAR), and two different resolutions (100 voxels and 200 voxels).

**Figure 2:**
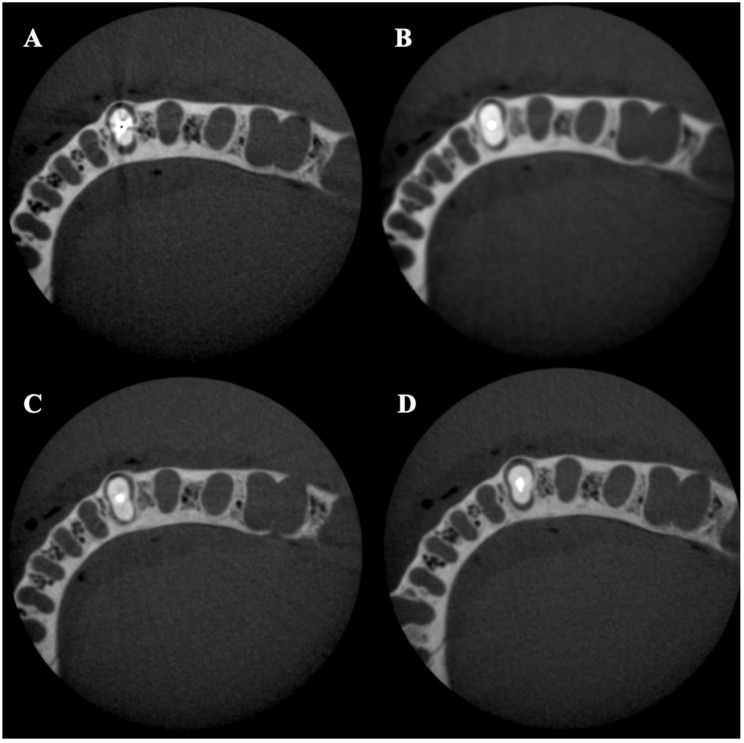
Axial slices of middle thirds for each post material obtained with no MAR, 90 kVp, and 100 voxels: (**A**) gold-plated post; (**B**) titanium alloy post; (**C**) quartz fiber (Bisco DT Light) post; (**D**) glass Fiber (Rely X) post.

A total of 480 CBCT scans were acquired using multi-slice digital imaging in communications in medicine (DICOM) format with 0.16 mm thickness. For each volume, two slices were selected at 2 and 4 mm from the cementoenamel junction (CEJ), representing the cervical and middle root thirds (Figure 2).

**Figure 3:**
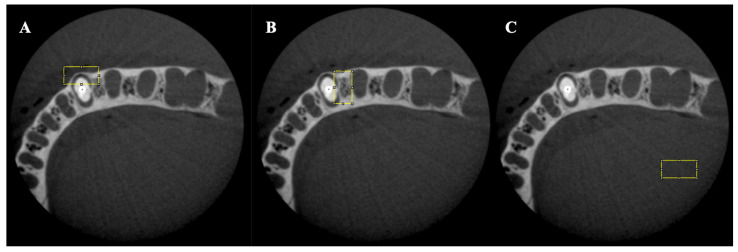
Area of interest for each middle and cervical third: (**A**) buccal area (width 60 × height 30); (**B**) interproximal area (width 30 × height 60); (**C**) control area (width 60 × height 30).

The analysis was performed using ImageJ software (National Institutes of Health, Bethesda, MD, USA). In each image, three areas were selected, and, for each area, the minimum, maximum, and mean gray values, as well as the standard deviation, were calculated. The first area was chosen as a rectangle (width 60 × height 30) in the buccal region of the root except for the post material. The second rectangular area (width 30 × height 60) was chosen in the interproximal area excluding the post material. Lastly, the third rectangular area (width 60 × height 30) was chosen as a control area away from the tooth and artifact toward the farthest edge of the volume for each slice (Figure 3). The rectangular areas included the straight lines of steaking artifacts and beam-hardening dark bands.

For each protocol, the dose area production (DAP) was saved and used as a reference to assess the amount of radiation provided. The DAP is automatically created and provided by the machine post exposure. The effective dose was calculated from the measured DAP value multiplied by a conversion coefficient. The kVp-dependent formula suggested by Batista et al. [18] was used to convert the DAP value to effective dose, which was expressed in mSv.

The effective dose from CBCT was calculated using the conversion formula suggested by Batista et al. [16]: E = (0:001453 × {kV} + 0:0118) × PKA, where kV is the tube voltage, E is the effective dose, and PKA is the DAP.

### 2.3. Statistical Analysis

Artifacts were analyzed using the standard analysis of variance technique, with all two-way, three-way, and four-way interactions among the four material types, two kVps, three MAR algorithms, and two voxel settings, and with tooth as a blocking factor (presumed not to interact with any of the machine settings or material). The Tukey multiple-comparison adjustment method was used to determine the factor combinations (material, kVp, voxel size, and MAR) with adequate evidence to be considered different from the others. A *p*-value < 0.05 was considered to indicate statistical significance.

## 3. Results

In Table 1, it can be seen at the 95% confidence level that each of the individual factors (material, kVp, voxel size, and MAR setting) had a statistically significant effect on the mean value of artifacts if all other factors were held constant. The interaction between material and MAR also had a statistically significant effect (*p* < 0.01). The remaining two-way interactions and all three-way and four-way interactions were not statistically significant (*p* > 0.05).

Tukey’s multiple-comparison adjustment method was used to detect which two-factor combinations had sufficient proof to be considered different from the others. Table 2 and Table 3 show the most meaningful comparisons, starting with the lowest artifact values for each comparison grouping.

There was a significant difference between the cervical and middle thirds of the root in terms of artifact creation, with more artifacts seen in the latter compared to the former (*p* < 0.05, Table 2).

Tukey’s multiple-comparison tests for material type, kVp, MAR, and voxel size are shown in Table 3. For both the cervical and the middle thirds of the root, the mean artifact value was highest for gold (Nordin), followed by titanium, quartz fiber (Bisco DT Light), and glass fiber (Rely X). The mean artifact value was higher for 90 kVp compared to 96 kVp. Similarly, the mean artifact value for no MAR was higher than that for high and medium MAR. No significant difference was found between medium and high MAR. Furthermore, the mean artifact value was higher for 100 voxels compared to 200 voxels. When all tables and comparisons were combined, it became evident that the best choice is to use glass or quartz fiber post at low resolution, with high MAR and 96 kVp.

The effective dose measured for each protocol and calculated from the DAP using a conversion formula demonstrated that the use of 100 voxels increased the effective dose, while 200 voxels resulted in less radiation. Additionally, 96 kVp increased the radiation dose compared to 90 kVp. The use of 90 and 96 kVp with 200 voxels and high MAR provided the least amount of radiation, while the fewest artifacts were produced using 96 kVp.

## 4. Discussion

The presence of posts within the root canal generates artifacts, which may hinder the examination of diagnosis of several endodontic conditions. Therefore, when CBCT is required in areas containing post materials, the clinician must be aware that these artifacts may alter the true diagnosis [8,19].

In particular, metal posts may cause scatter, blooming, and noise, which can affect the image quality and hinder the identification of cracks, additional root canals, procedural errors, or lesions of the endodontically treated or adjacent teeth in clinical conditions [20].

In the present study, along with the gold-plated and titanium posts, two different types of silica fiber posts were used. Rely-X fiber post has a composition of glass fiber (60–70%), epoxy resin matrix (30–40%), and an additional zirconia (Zr) filler component. DT-Light Post, on the other hand, is composed of epoxy matrix (38%) and quartz fiber (62%). Having similar components with similar percentages, their radiopacity values were also shown to be similar [21,22]. According to the atomic numbers of materials and fillers in the compositions, gold-plated posts presented the highest number of artifacts as expected. When other factors are not considered, glass fiber and quartz fiber posts should be used to avoid artifacts for future radiologic examination.

There have been numerous material studies on artifact evaluation in various CBCT settings including FOV, kVp, mA, voxel, and MAR.

De Rezende Barbosa et al. [23] reported that gold posts reduced the overall CBCT diagnostic ability regardless of the use of an artifact reduction algorithm. On the other hand, fiber posts produced fewer image artifacts with a more uniform energy absorption [24]. Similarly, quartz fiber and glass fiber posts demonstrated fewer artifacts in our study. The best choice was found to be glass or quartz fiber posts for artifact reduction in the present study.

De Martin E Silva et al. [25] assessed the influence of applying filters (sharpen and hard) and voxel sizes (0.25 mm and 0.30 mm) on teeth with and without one type of metal post in CBCT images. The authors observed no differences between filters, whereas images obtained with a 0.25 mm voxel size were more accurate than those obtained with a 0.30 mm voxel. The results of the study demonstrated that metal posts and voxel size adversely affected the exact diagnosis, while applying filters did not influence the diagnosis.

Fontenele et al. [26] investigated the impact of direction and composition of metallic post materials on the expression of artifacts. Their results showed that the mandibular posterior region had significantly lower gray values than the anterior region, and the silver–palladium group exhibited the highest expression of beam hardening artifacts in the posterior region of the mandible.

Pinto et al. [27] examined the influence of exposure settings (kVp, mA) and the composition of the root canal (unrestored, gutta-percha, metallic post, and fiberglass post) on the detection of vertical root fractures. The study revealed that, within the same material group, there were no significant differences among different combinations of kVp and mA parameters. However, in the metallic post group, due to the potential decrease in the kVp/mA settings, vertical root detection was deteriorated because of the greater artifact formation.

Helvacıoglu-Yigit et al. [4] stated that the use of 96 kVp with MAR and low resolution diminished the artifacts in the image and concurrently achieved the lowest effective dose. In the current study, the mean artifact value was higher when 90 kVp without MAR was used. This finding is consistent with previous studies [4,27].

Lira de Farias Freitas et al. [16] evaluated artifacts caused by post materials for the cervical, middle, and apical root thirds, and they concluded that hyperdense artifacts were more evident in the cervical third than in the apical third of the teeth. They explained this difference according to the greater proportion of the tooth area in the apical third compared to the cervical third. In the present study, buccal and interproximal rectangular areas including dentine and alveolar bone but not post materials were considered for gray value calculation in the cervical and middle thirds, and more artifacts were seen in the middle third compared to the cervical third. We concluded that, according to the difference in remaining dentine proportion for the cervical and middle thirds, although the total area of the tooth decreases while the post area decreases, the remaining dentine area is lesser in the middle third, especially in the interproximal areas. Therefore, decreasing the remaining dentine around the post material may hinder diagnosis because of artifacts.

Dose optimization utilizing exposure parameters below the manufacturer’s default settings should always be explored, especially for young patients. Since exposure parameters, effective dose, and image quality are related, it is crucial to choose the exposure parameters to ensure the best image quality while lowering the effective dose to a minimum level according to the “as low as diagnostically acceptable” principle.

In the current study, the use of 90 and 96 kVp with 200 voxel size and high MAR provided the least amount of radiation, while the fewest artifacts were produced using 96 kVp, making 96 kVp, high MAR, and low resolution the best clinically applicable protocol in the Planmeca Promax for assessment of a tooth with post-restoration or a tooth or region close to a tooth with post-restoration.

In our study, we calculated the effective dose from the DAP using a conversion formula. However, in clinical conditions, the effective dose, E, is calculated as follows:

Effective dose (E) = ∑absorbed dose × radiation weighting factor (WR) × tissue weighting factor (WT).

The tissue weighting factors are needed because, even if the equivalent dose is the same, different organs have different levels of sensitivity to radiation. There are two types of personal dosimeters; pocket dosimeters are usually used for monitoring over a shorter timeframe, whereas dosimeter badges are used to measure cumulative doses over periods of weeks or months. The more commonly used dosimeter badges are based on thermoluminescent dosimeters (TLDs) and the optically stimulated luminescence of materials such as Al_2_O_3_.

This study had some limitations. The first limitation was its in vitro design. In actual clinical cases, various interferences (e.g., metallic artifacts due to prosthesis, implant location, and the risk of patient movement at the time of acquisition) may handicap the diagnosis. The second limitation was the absence of a crown or filling restoration in the upper structures of the post materials. No restoration parameter was added to standardize the artifact calculation, but this is obviously not the case in the clinical situation. The final limitation was the use of only one CBCT unit. Therefore, further in vivo studies are necessary with different types of machines.

## 5. Conclusions

In conclusion, glass or quartz fiber posts caused fewer artifacts than gold and titanium posts; hence, their use is considered more adequate as a dental post material to generate images that are more suitable to detect fine details. Furthermore, more artifacts were seen in the middle third compared to the cervical third. The use of low resolution, high MAR, and 96 kVp led to the lowest effective doses, as well as the image with the fewest artifacts. Various CBCT machines use diverse methods to reduce metal artifacts; further studies investigating these methods and their application in preclinical and clinical settings are needed.

## Figures and Tables

**Table 1:** Abbreviated analysis of variance table for artifacts (*R*^2^ = 0.995).

Source	df	F-Ratio	*p*-Value
Model	48	1860.81	<0.0001
Type	3	35.31	<0.0001
KVP	1	10.67	0.0012
MAR	2	11.74	<0.0001
Voxel	1	47.67	<0.0001
Type × KVP	3	0.09	0.9675
Type × MAR	6	6.36	<0.0001
Type × Voxel	3	0.76	0.5197
KVP × MAR	2	0.52	0.5973
KVP × Voxel	1	3.81	0.0516
MAR × Voxel	2	0.25	0.7766
Type × KVP × MAR	6	0.76	0.6048
Type × KVP × Voxel	3	1.40	0.2415
Type × MAR × Voxel	6	0.29	0.9429
KVP × MAR × Voxel	2	0.02	0.9831
Type × KVP × MAR × Voxel	6	0.88	0.5096
Error	432		
Total	480		

**Table 2:** Artifact comparison by region.

Region	Artifact		*p*-Value
	Mean	SD	
**Cervical third**	156.913542	13.7407142	-
**Middle Third**	165.252083	16.0284193	<0.05

**Table 3:** Artifact comparison by material type, KVP, MAR, and voxel size.

	**Material Type**	**Mean Value of Artifact**	***p*-Value**
Cervical third	Glass fiber (Rely X)	150.78	-
	Quartz fiber (Bisco DT Light)	153	>0.05
	Titanium (Unimetric)	159.33	<0.01
	Gold (Nordin)	161.08	<0.01
Middle third	Glass fiber (Rely X)	161	-
	Quartz fiber (Bisco DT Light)	162	>0.05
	Titanium (Unimetric)	166	<0.05
	Gold (Nordin)	171	<0.01
	**kVP**	**Mean Value of Artifact**	***p*-Value**
Cervical third	96 kVp	155.2	-
	90 kVp	158.63	<0.05
Middle third	96 kVp	163.33	-
	90 kVp	167.16	<0.05
	**MAR**	**Mean Value of Artifact**	***p*-Value**
Cervical third	High	154.6	-
	Medium	155.67	>0.05
	No	160.46	<0.05
Middle third	High	163.59	-
	Medium	164.45	>0.05
	No	167.71	<0.05
	**Voxel**	**Mean Value of Artifact**	***p*-Value**
Cervical third	100	160.54	-
	200	153.28	<0.05
Middle third	100	170.47	-
	200	160.03	<0.05

## Data Availability

The data in this study are available upon request from the corresponding author.
